# SEIPIN: A Key Factor for Nuclear Lipid Droplet Generation and Lipid Homeostasis

**DOI:** 10.3390/ijms21218208

**Published:** 2020-11-02

**Authors:** Yi Jin, Yanjie Tan, Pengxiang Zhao, Zhuqing Ren

**Affiliations:** 1 Key Laboratory of Agriculture Animal Genetics, Breeding and Reproduction of the Ministry of Education & Key Laboratory of Swine Genetics and Breeding of the Ministry of Agriculture and Rural Affairs, College of Animal Science, Huazhong Agricultural University, Wuhan 430070, Hubei, China; hyj_1900@webmail.hzau.edu.cn (Y.J.); tanyanjie@webmail.hzau.edu.cn (Y.T.); pengxiang@webmail.hzau.edu.cn (P.Z.); 2 Bio-Medical Center of Huazhong Agricultural University, Wuhan 430070, Hubei, China; 3 Institute of Biomedical Sciences, Key Laboratory of Animal Resistance Biology of Shandong Province, College of Life Sciences, Shandong Normal University, Jinan 250014, Shandong, China

**Keywords:** nuclear, lipid droplet, SEIPIN, lipid homeostasis

## Abstract

Lipid homeostasis is essential for normal cell physiology. Generally, lipids are stored in a lipid droplet (LD), a ubiquitous organelle consisting of a neutral lipid core and a single layer of phospholipid membrane. It is thought that LDs are generated from the endoplasmic reticulum and then released into the cytosol. Recent studies indicate that LDs can exist in the nucleus, where they play an important role in the maintenance of cell phospholipid homeostasis. However, the details of nuclear lipid droplet (nLD) generation have not yet been clearly characterized. SEIPIN is a nonenzymatic protein encoded by the Berardinelli-Seip congenital lipodystrophy type 2 (*BSCL2*) gene. It is associated with lipodystrophy diseases. Many recent studies have indicated that SEIPIN is essential for LDs generation. Here, we review much of this research in an attempt to explain the role of SEIPIN in nLD generation. From an integrative perspective, we conclude by proposing a theoretical model to explain how SEIPIN might participate in maintaining homeostasis of lipid metabolism.

## 1. Background

Lipids are an important component of life, playing an important role in the formation of cellular membranes and energy production for numerous cell biological processes [1]. To store extra lipids for further utilization, bacteria and eukaryotic cells have developed a specialized organelle, termed a lipid droplet (LD). LDs are composed of an esterified neutral lipid core of triacylglycerols (TAG) and sterol esters (SEs), a monolayer phospholipid membrane, and some proteins. LDs allow the cell to buffer fluctuations in lipid availability [2,3,4]. For example, the cell can utilize LDs to generate energy during starvation. LDs can also protect the cell from lipotoxicity during the intake of a large amount of fatty acids (FAs) [5].

LDs are now recognized as being highly dynamic [6,7], multifunctional organelles that are involved in many cell biological processes [2]. For example, the LD content responds to changes in metabolic conditions [5,8]. Furthermore, LDs can provide different lipid components such as phospholipids and FAs for cellular membrane formation during proliferation [1]. De novo LD biogenesis begins at the endoplasm reticulum (ER) though they are released into the cytoplasm [3,4]. However, recent studies indicate that LDs are also present in the nuclear. It is not possible that LDs enter the nuclear, because the diameter of LDs are far larger than nuclear pore. Therefore, the ER might not be the only site for LD biogenesis [9,10]. Nuclear LDs (nLDs) are considered to play an important role in maintaining nuclear lipid homeostasis and regulating cellular phospholipid levels [10]. However, the mechanism of nLD generation has not yet been clearly characterized.

SEIPIN is a protein encoded by the Berardinelli-Seip congenital lipodystrophy type 2 gene (*BSCL2*) and is located in the ER membrane [11]. *BSCL2* was identified by Magre et al. in 2001, who found that mutations in a specific locus of the 11q13 chromosome were associated with Berardinelli-Seip congenital lipodystrophy type 2 [12]. Therefore, SEIPIN was recognized as an important regulator involved in lipodystrophy. Since then, SEIPIN has attracted the attention of numerous researchers who have investigated its functions as a regulatory mechanism in adipogenesis. Nineteen years later, SEIPIN is now recognized as an important factor during LD biogenesis [13,14,15]. SEIPIN knockout greatly affects LD morphology and content [16,17]. SEIPIN is highly conserved among species and regulates LD production in yeast, nematodes, and plants. Fld1p is a homolog of human SEIPIN in yeast, and Fld1p deletion in yeast results in super-large LDs, and the expression of human-derived SEIPIN rescues the Fld1p-induced phenotype [18]. SEIP-1 in nematodes is a homolog of human SEIPIN, which is important for the regulation of LD production and lipid homeostasis during embryogenesis and is required for the permeability barrier for eggshell synthesis [19]. *Arabidopsis* has three homologs of the human SEIPIN gene: SEIPIN1, SEIPIN2, and SEIPIN3. These three genes differentially regulate the number and size of LDs and regulate neutral lipid storage in plants [20].

In this review, we integrated the results of several papers, and found evidence for the regulation of SEIPIN in nLD generation. Moreover, we propose a theoretical model to explain the role of SEIPIN in maintaining homeostasis of lipid metabolism.

## 2. Biogenesis, Distribution, and Function of LDs

### 2.1. LD Biogenesis

According to a widely accepted model, LD biogenesis can be divided into four steps, including neutral lipid synthesis within the ER, formation of an oil lens in the ER membrane, budding and nascent LD formation, and LD growth and expansion [3]. At first, neutral lipids such as TAG and SE are de novo synthesized in the ER, mainly due to terminal enzymes located on the ER membrane [3,21,22]. The ER surface contains various lipid synthesis enzymes: acyl-CoA synthetase long chain family member 3 (ACSL3), stearoyl-CoA desaturase (SCD1), glycerol-3-phosphate acyltransferases (GPATs), acylglycerol-3-phosphate-o-acyltransferases, phosphatidate phosphatases, and diacylglycerol-o-acyltransferases (DGATs) [3,4]. Cells take in or de novo synthesize FAs, and FAs and glycerin can then be converted into esterified lipids by these synthases. The synthesized neutral lipids move freely within the ER bilayer. As neutral lipids accumulate within the ER bilayer, they merge into a structure termed the “lens” via a biophysical process [23,24]. As the lipid lens grows, it finally buds into the cytosol. The process of budding itself can be thought of as a dewetting process in which a TG lens is formed and converted to a bud on the ER surface [3]. These newly formed LDs interact with the ER and complete the transport of lipids from the ER to LDs through a bridge structure [25,26]. In addition, TG synthases, such as GPAT4 and DGAT2, can be transferred to the surface of LDs from the ER via direct contact. Therefore, TGs are synthesized on the surface of the LDs and then transferred to the neutral lipid core [27,28].

### 2.2. LD Distribution

**Figure 1:**
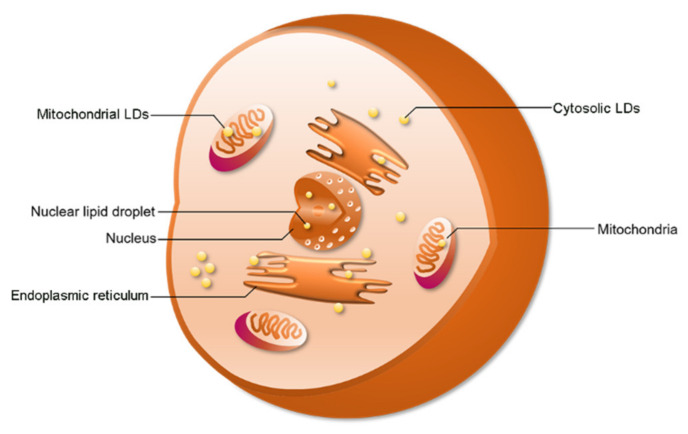
The distribution of lipid droplets (LDs). LDs have been found in the cytosol, nucleus, and mitochondria. Cytosolic LDs account for a large proportion of total LDs.

Cytosolic LDs (cLDs) are common in specific cell types or under specific physiological conditions. Interestingly, recent studies indicate that LDs can also be found in the nucleus and mitochondria (Figure 1). In 2006, Mate et al., studied the lipid components in nuclei [29]. They investigated the lipid composition of membrane-depleted rat liver nuclei and found that the fatty acid composition and phosphatidylcholine molecular species distribution in the nucleus were similar to that of the whole nucleus, mitochondria, and homogenate of cells [29]. However, the researchers did not know how and where the nuclear lipids were stored. LDs allow the cell to store extra lipids. In 2012, the investigators found LDs in the nucleus, as expected [30]. When investigating the composition and organization of neutral nuclear lipids, Layerenza et al. found that these neutral lipids were organized into nonpolar domains in the form of nLDs [30]. A recent study showed that LDs are also found in mitochondria [31]. Moreover, there are also reports of LD formation in plastids in algae and plants [32]. Therefore, these results suggest that LDs are generated by other organelles.

### 2.3. Function of cLDs and nLDs

Besides storing extra esterified lipids, LDs have many other functions such as protection against cellular stress [33], responding to invasion by bacteria [34,35] and viruses [36,37], regulation of immune-related processes [38], storage of proteins such as histones [39], and protein quality control [40]. Under stress conditions, cells can be adversely affected by reactive oxygen species (ROS). The membrane lipids and proteins can be oxidized by ROS. LDs can decrease ROS damage by absorbing 4-hydroxynonenal (HNE, a product of lipid oxidation that causes protein damage) from the cell membrane [41,42]. Moreover, LDs can esterify and store extra FAs when cells are in an FA-rich environment [33,43,44], which can protect cells against lipotoxicity (since an excessive amount of extra FAs induces cell apoptosis). Invasion by bacteria and viruses can be recognized as a stress condition for cells. LDs play an important role in the assembly and maturation of the hepatitis C virus protein core [37]. Furthermore, the dengue virus protein NS4A/B can bind to the LD-surface protein Ancient Ubiquitous Protein 1 (AUP1) and promote LD degradation by autophagy [36]. The energy generated by the decomposition of LDs is very important for the replication and infection of dengue virus [36]. It was found that in LDs, cyclooxygenase (COX)-2, a key enzyme in prostaglandin E2 (PGE2) synthesis, is recruited to promote PGE2 synthesis [45]. High LD content is associated with high PGE2 synthesis [46]. PGE2 is an important immunosuppressive factor that inhibits the proliferation of T cells [47,48]. Moreover, a recent study identified the relationship between LD accumulation and airway inflammation, e.g., asthma caused by chronic activation of innate lymphoid cells [38]. The LDs in pathogenic group 2 innate lymphoid cells provide energy and phospholipids for cell proliferation, which leads to the chronic activation of innate lymphoid cells [38].

The function of cLDs is well understood, but the function of nLDs is not completely clear. Some studies suggest that nLDs are organized into domains similar to those of cLDs [30]. Electron microscopy has revealed that nLDs constitute specific subdomains of the nucleus [49], which could be involved in nuclear lipid homeostasis. Wang et al., suggested that nLDs might result as a response of cells to stress. To test this, they treated mice with perfluorooctanoic acid (PFOA), a synthetic perfluorinated compound, which is considered to be a general pollutant in the environment [50]. They found that LDs accumulated in nuclei of hepatocytes. Hence, PFOA toxicity might have been the reason for the observed generation of nLDs [50]. A recent study from Fujimoto’s group supports the relationship between nLDs and stress [10]. They found that more nLDs were generated during ER stress [10]. As a feedback process, nLD accumulation promotes phosphatidylcholine synthesis by increasing CDP-choline diacylglycerol phosphotransferase α (CCTα) recruitment [10]. In bacteria, LDs can bind to DNA to prevent DNA damage and promote the bacteria survival rates during stress conditions [51,52]. In summary, nLDs might be important for nuclear lipid homeostasis, cellular phospholipid homeostasis, and stress responses.

## 3. Structure and Function of SEIPIN

### 3.1. SEIPIN Structure

**Figure 2:**
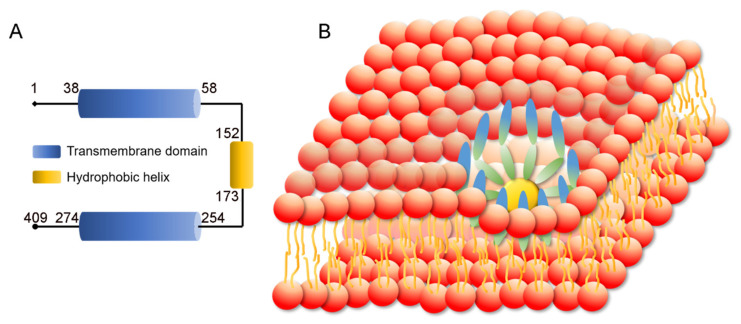
Structure of Berardinelli-Seip congenital lipodystrophy type 2 protein (SEIPIN). (**A**) SEIPIN consists of two transmembrane domains and a hydrophobic helix domain. (**B**) Human SEIPIN is a ring-shaped trans-membrane protein complex, which consists of 11 subunits.

The SEIPIN protein is encoded by the *BSCL2* gene. According to NCBI and UniProt databanks, three *BSCL2* transcription variants generate three SEIPIN isoforms. SEIPIN isoform s1, 2, and 3 have 409, 287, and 462 amino acids, respectively. Isoform 1 is considered the canonical isotype according to UniProt [53]. The total weight of SEIPIN is 508.18 kDa, representing a residue count of 4499. SEIPIN is a transmembrane protein with two hydrophobic helices, with the N and C termini facing the cytosol. The SEIPIN protein forms a large protein complex. The yeast SEIPIN complex consists of 9 subunits. Single particle electron microscopy indicated that the large complex has the shape of a donut with a central cavity [54]. A recent study revealed that the human SEIPIN ring-shaped protein complex consists of 11 subunits [55] (Figure 2). High resolution atomic models of the conserved luminal domains revealed hydrophobic α-helices at the inner rim of the oligomeric ring which likely bind to the ER membrane [55,56].

### 3.2. SEIPIN Function

SEIPIN has a key role in adipose tissue homeostasis. Studies in a *BSCL2* knockout mouse model showed that SEIPIN deficiency causes severe and consistent lipodystrophy with a dramatic loss of fat mass [57,58]. Furthermore, production from adipose tissues, such as adipokines, leptin and adiponectin, is also decreased in *BSCL2* knockout mice [57,58]. The histological analysis indicated that many adipocytes were immature, and the LD content was reduced in both white and brown adipose tissues [57,58]. Moreover, the expression of several marker genes involved in terminal adipocyte differentiation, such as adiponectin and fatty acid binding protein (FABP4), were altered in *BSCL2* knockout mice [59] and, additionally, the expression levels of preadipocyte marker, Pref1, were decreased in *BSCL2* knockout mice [58]. These results indicate that SEIPIN plays an important role in adipose tissue maintenance.

SEIPIN is also involved in adipocyte differentiation. During adipogenesis, SEIPIN expression is increased [60]. Knockdown of SEIPIN can inhibit the terminal differentiation of adipocytes [60]. SEIPIN deficiency does not affect commitment of the preadipocyte into an adipocyte but mainly affects the terminal maturation of adipocytes [58,59].

One of the most important functions of SEIPIN is as a mediator or “lipidic bridge” connecting LDs to the ER. SEIPIN has been found at LD–ER contact sites in numerous studies [14,17,28,61]. According to the SEIPIN ring-shaped structure, the LD and ER can be connected by the SEIPIN ring. This “lipidic bridge” is essential for LD growth. On the one hand, the esterified lipids within the bilayer phospholipid membrane of the ER can be transferred to nascent LDs, which is important for LD expansion [28]. Moreover, cytosolic LDs can also come into contact with the ER again and take in neutral lipids within the ER membrane [3,4,28]. On the other hand, several lipid synthesis proteins, such as GPAT4 and DGAT2, on the ER membrane can be transferred to LDs through the “lipidic bridge”, which is essential for LD growth and expansion because these lipid synthases can catalyze the esterification of free FAs into neutral lipids, allowing them to be stored in LDs [28].

## 4. The Role of SEIPIN in cLD and nLD Biogenesis

The role of SEIPIN in cLDs has been well studied. SEIPIN knockout results in dramatic changes in the number and morphology of LDs. The number of LDs is strongly decreased by SEIPIN deficiency, but giant LDs can usually be observed in cells with SEIPIN knockout. During LD biogenesis, endogenous SEIPIN forms patches in the ER, and these patches move rapidly along ER tubules [28]. When some patches of SEIPIN encounter sites of neutral lipid accumulation, they co- localize with these neutral lipid collections, where the initial growth of nascent LDs is then promoted [28]. Therefore, SEIPIN functions in the initial steps of LD formation either by helping to generate nascent LDs or by facilitating their growth and expansion [17,28]. Moreover, SEIPIN deficiency was found to induce altered LD surface proteome composition [17,62,63]. SEIPIN deletion leads to abnormal transfer of some TAG synthase enzymes to LDs during early LD formation, resulting in the formation of large LDs [28]. LDs and the ER stay in contact even when SEIPIN is absent [17,28], indicating other proteins involved in definition of the initial budding and growth step of LD formation.

**Figure 3:**
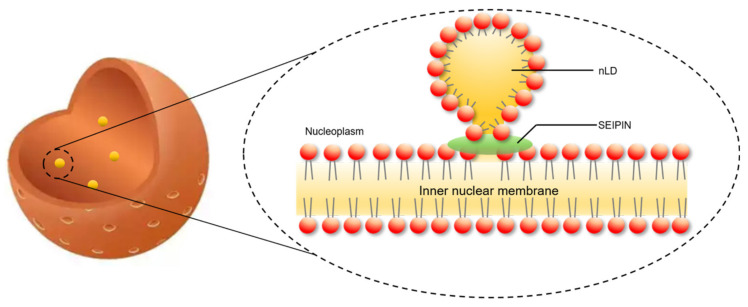
SEIPIN is involved in nuclear lipid droplets generation. SEIPIN mediated the generation of a lipidic bridge, which connects the nascent nLD and the inner nuclear membrane.

**Figure 4:**
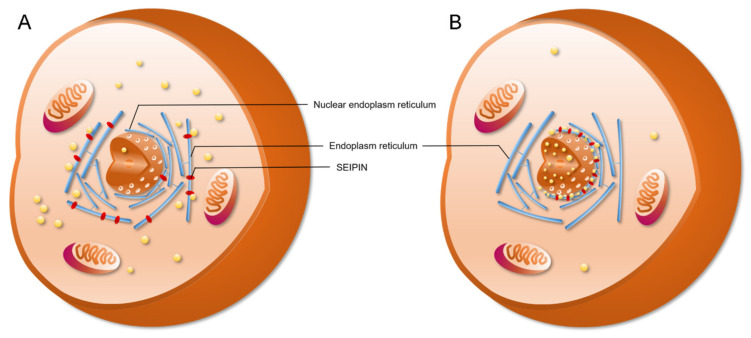
SEIPIN localization affects nuclear lipid droplets generation. (**A**) SEIPIN is normally an endoplasm reticulum (ER)-localized protein. SEIPIN localized at the ER induces the generation of cLDs. (**B**) When expressing nuclear-ER-trapped SEIPIN in SEIPIN knockout cells, more nLDs were observed.

SEIPIN also contributes to the generation of nLDs. Recent studies revealed that SEIPIN participates in the biogenesis of nLDs [9,14]. Romanauska et al. found that SEIPIN promoted the formation of inner nuclear membrane–nLD membrane bridges [9] (Figure 3). They found that SEIPIN co-localized with Nup60, an inner nuclear membrane marker [9], at the inner nuclear membrane. Moreover, fluorescence signals corresponding to the interaction of SEIPIN with Nup60 were also found at nLDs [9]. The deletion of SEIPIN resulted in the disappearance of the defined membrane bridges [9]. The nLDs were also found to adhere closely to the inner nuclear membrane [9]. Moreover, cells displayed irregular periplasmic cavities with SEIPIN deletion. These results indicate that SEIPIN affects the formation of membrane bridges with nLDs and the architecture of the periplasmic space during nLD generation [9]. Another interesting result was reported by Salo et al. [14]. They investigated the ER–LD contacts and the delivery of triglycerides from the ER to LDs [14]. They creatively constructed a SEIPIN-NE (nuclear endoplasmic reticulum) trap system. Briefly, two expression vectors were constructed. One was SEIPIN-EGFP expression vector, and the other was a SUN2-KASH2 trimer vector, which contained a GFP-nanobody. SUN domain-containing protein 2 (SUN2) and Nesprin-2 (KASH2) are two well-known NE markers. When SEIPIN-EGFP is expressed at the ER, the GFP binds to the GFP-nanobody thereby trapping the SEIPIN protein at the NE [14]. Interestingly, the location of SEIPIN at the NE induced nLD generation [14] (Figure 4). Although Salo et al. did not discuss this phenotype in their study [14], the result was very meaningful for the study of nLD generation.

## 5. The Role of SEIPIN Partners in Maintaining Cell Lipid Homeostasis

**Figure 5:**
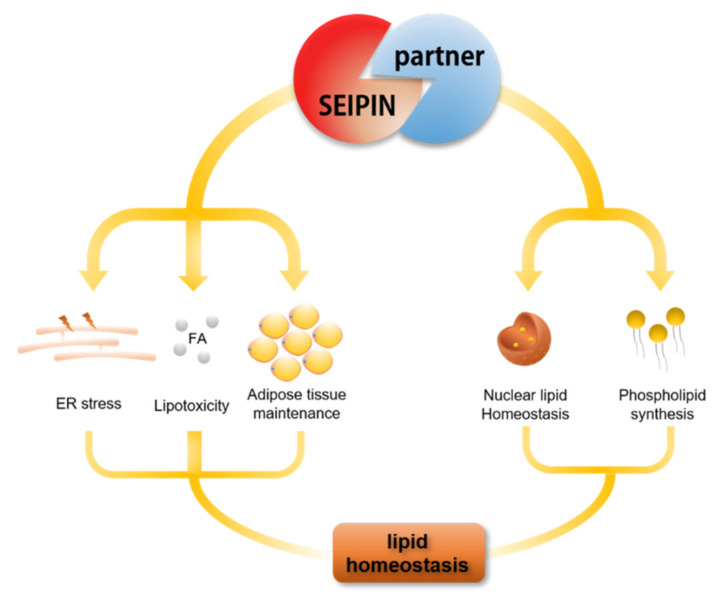
SEIPIN and partner proteins (that interact with SEIPIN) play an important role in cell lipid homeostasis.

SEIPIN is very important for LD biogenesis, adipocyte differentiation, and adipose tissue homeostasis. Therefore, there is no doubt that SEIPIN maintains lipid homeostasis. However, many partners interacting with SEIPIN are also involved in the lipid metabolism process, such as LD assembly factor 1 (LDAF1) [64], Pex30 (containing certain peroxisomal membrane proteins) [15], SERCA (sarco/endoplasmic reticulum Ca^2+^-ATPase) [65] and promethin/TMEM159 [66]. Models of the SEIPIN structure show a hydrophobic helix of each subunit of the oligomeric ring positioned in the luminal leaflet of the ER [55,67]. The hydrophobic helix is a part of the highly conserved luminal domain of SEIPIN [68]. Chung et al. found the hydrophobic helix of SEIPIN (152–173 aa) has strong sequence conservation among species from worm to human [66]. Furthermore, they investigated the proteins interacting with the hydrophobic helix of SEIPIN and found that LDAF1, as an interaction partner of SEIPIN, regulates LD biogenesis [66]. LDAF1 and SEIPIN form an oligomeric complex which is the site of LD formation, and LDAF1 then dissociates from SEIPIN and moves to the growing LD surface [66]. Moreover, LDAF1 can facilitate LD formation even at low ER triglyceride concentrations [66]. Besides the findings with LDAF1, Wang et al. found that Pex30 formed a complex with SEIPIN, which plays an important role in LD and peroxisome biogenesis in the ER [15]. They found that SEIPIN and Pex30 stabilized ER domains permissive for budding, but deletion of SEIPIN or Pex30 induced toxic TG accumulation [15]. Bi et al., found SERCA, an ER calcium pump that is solely responsible for transporting cytosolic calcium into the ER lumen, physically interacted with SEIPIN and strongly affected fat storage in *Drosophila* fat cells [65]. They also found that deficiency of either SEIPIN or SERCA reduced fat storage but increased fatty acid oxidation [65]. Promethin is a newly identified SEIPIN partner protein that is upregulated during adipogenesis [66]. It is recruited and physically interacts with SEIPIN to promote re-localization to the ER [66]. Besides the SEIPIN partner proteins discussed above, there are also many partner proteins that interact with SEIPIN physically, such as phosphatidic acid phosphatase (LIPIN1) [69], acylglycerol-phosphate acyltransferase (AGPAT2) [70], glycerol-3-phosphate acyltransferase (GPAT3) [71], steaoryl-CoA desaturase 1 (SCD1) [72], reticulon-like protein (REEP1) [73], and the adaptor protein 14-3-3β [74]. Therefore, SEIPIN together with its partner proteins plays an essential role in maintaining cellular lipid homeostasis (Figure 5).

## 6. The Potential Biological Significance of SEIPIN in Regulating nLD Generation

Since SEIPIN regulates the generation of nLDs, what is the biological significance of this phenotype? Although the biological functions of nLDs are not totally understood, the biogenesis of nLDs can be activated by stress conditions, such as oleic acid treatment, tunicamycin treatment, and PFOA exposure, which can induce lipotoxicity, ER stress, and cytotoxicity, respectively. Therefore, we speculate that the generation of nLDs is a stress response. In *S. cerevisiae*, Opi (transcription factor) translocation into the nucleus promoted the formation of nLDs and repressed the expression of lipid synthesis-related genes, thereby suppressing the formation of cLDs [9]. The formation of nLDs can negatively regulate the formation of cLDs. Furthermore, recruitment of the key phospholipid synthesis enzyme, CCTα, to the surface of nLD can further activate phospholipid synthesis, which is important for maintaining membrane homeostasis during cellular stress [10]. Therefore, the production of nLD may be an important part of resisting cellular stress.

## 7. Relationship Between LDs and Human Diseases

In order to store excess lipids, such as sterols or FAs, cells esterify these lipids to form neutral lipids and package them into cytosolic LDs. In humans, adipocytes in white and brown adipose tissue are used exclusively to store lipids in LDs. However, other cells also store lipids in LDs, including hepatocytes, intestinal cells, macrophages, and adrenal cortex cells. Having too many or too few LDs can easily lead to disease [75]. Human lipodystrophy is the clinical manifestation of systemic (congenital systemic lipodystrophy, CGL) or partial (familial partial lipodystrophy, FPL) body fat loss [76,77,78]. Lipid malnutrition causes serious changes in systemic energy metabolism, which is often related to metabolic disorders such as insulin resistance, liver steatosis, and hypertension. Failure to store TGS in white adipose tissue causes fat storage in other tissues and tissue lipotoxicity. Lack of white adipose tissue leads to leptin deficiency and related metabolic defects, such as insulin resistance [79,80]. In addition, cachexia is an acute wasting disease. The weight of patients with cancer cachexia drops sharply, and adipose tissue is rapidly lost in the early stage [81]. Increased lipolysis is a key factor in cachexia, and patients with cachexia have elevated blood glycerol and FAs [82]. Excessive LDs often lead to changes in system metabolism, including metabolic syndrome, which can increase the risk of type 2 diabetes, steatohepatitis, and coronary heart disease. In the case of adipocyte hypertrophy, the secretion of adipokines is insufficient for maintaining insulin sensitivity, and the secretion of pro-inflammatory cytokines such as monocyte chemoattractant protein-1 (MCP-1) and tumor necrosis factor-α (TNF-α) thereby results in the induction of macrophage infiltration and inflammation [83]. Genetically speaking, mutations of genes encoding adipose triglyceride lipase (ATGL) and its cofactor CGI-58 can cause neutral lipid deposition disease [84]. The mutation of CGI-58 is related to ichthyosis, a defect in the skin’s permeability barrier, and ATGL mutation can cause severe cardiomyopathy and systemic lipid accumulation in humans and mice [84,85]. In addition, the lack of ATGL in the heart can reduce the expression of PPARα/peroxisome proliferator-activated receptor-γ coactivator 1(PGC-1α), leading to myocardial mitochondrial dysfunction and lipid accumulation, leading to heart failure [86]. Fatty liver disease and LD metabolism syndrome in liver disease are often accompanied by nonalcoholic fatty liver disease and the accumulation of triglyceride-containing LDs in liver cells. Upregulation of ADRP levels [87], PNPLA3/ADI polymorphisms [88], liver lipase (LIPC/HTGL) and lysophospholipase-like protein 1 (LYPLAL1) gene mutations [89,90], and DGAT2 enzymes [91] involved in TG synthesis are also associated with the risk of liver steatosis. In addition, blocking the secretion of very low-density lipoproteins can lead to nonalcoholic fatty liver [92]. The accumulation of cholesterol esters in the arteries is closely related to atherosclerosis, and may lead to myocardial infarction, stroke, or sudden cardiac death. Cholesterol esters in the arteries are mainly stored in the low-density lipoproteins (LDLs) of the foam cells of macrophages [93]. Observation by microscope revealed that the lipoproteins containing atherosclerotic apolipoprotein B (low-density lipoproteins, chylomicrons) and very low-density lipoprotein residues accumulate under the endothelium and are taken up by macrophages, which contain large amounts of cholesterol. Macrophages that accumulate a large amount of cholesteryl ester become foam cells [94]. Esterification of cholesterol in foam cells seems to be protective because free cholesterol is toxic to cells and is pro-inflammatory [94]. The formation and storage of cholesterol esters in macrophages may provide buffering capacity until cholesterol can be removed from the arterial wall through cholesterol efflux and reverse cholesterol transport [93]. If cholesterol accumulates beyond the clearance mechanism, inflammation and pathology progress, similar to wounds, leading to plaque formation, rupture, and thrombosis [95]. Through an in-depth understanding of LD biology, important factors in LD generation and decomposition pathways can be artificially regulated, such as triglyceride synthase and DGAT2 in the LD generation pathway, and lipases such as ATGL and HSL in the LD decomposition pathway. In addition, the number of LDs can be adjusted such that they are maintained in the normal range, thereby reducing the risk of diseases caused by too many or too few LDs.

At present, diseases related to LDs in the nucleus have not yet been reported. Based on the current research of LDs in the nucleus, the cell is observed to produce nuclear LDs when the external environment of the cell changes, such as when there is an increase in free fatty acid content [96,97], oxidative stress, or the presence of exogenous toxic substances (PFOA) [50]. In addition, intranuclear LDs are not common in cells with the exception of hepatocytes [29,98]. Romanauska et al., pointed out that the production of LDs in the nucleus can regulate the synthesis of cytoplasmic LDs in yeast [9]. Following the formation of LDs in the nucleus, Opi1, an important transcription factor regulating lipid synthesis, is recruited to the surface, thereby inhibiting the lipid synthesis pathway [9]. In addition, Sołtysik et al., showed that LDs in the hepatocytes can recruit the phospholipid synthase CCTα, thereby activating phospholipid synthesis and maintaining the stability of cell membrane components [10]. Therefore, we speculate here that LDs in the nucleus probably play a role in the storage of cell lipids and cell stress. When LDs in the nucleus cannot be generated, cytoplasmic LDs are lacking a feedback regulation pathway which leads to an abnormal increase in the number of LDs. Moreover, defective formation of LDs in the nucleus may lead to a decrease in the ability of cells to resist stress and reduce cell adaptability.

## 8. Outlook

SEIPIN is a key player in lipid storage, but the molecular mechanisms involved in regulating lipid homeostasis are not totally understood. Here, we reviewed recent studies to clarify the role of SEIPIN in the regulation of LD biogenesis, especially nLDs, the new kind of LD [99]. Moreover, we also reviewed the regulatory role of SEIPIN and its partner proteins in controlling lipid storage. The recent interesting results give us a new view on SEIPIN.

SEIPIN and its partner proteins form a complex which promotes TG accumulation and the growth of LDs in addition to also being a site for LD formation. Therefore, the location of SEIPIN is essential for the position of LD generation. Although the mechanism of nLD generation is not totally clear, SEIPIN plays an important role in connecting nLDs and the inner nuclear membrane and contributes to formation of the “lipidic bridge”, which is considered to be important for LD growth and expansion. The function of nLDs is also largely unknown, but nLDs are likely to participate in storing nuclear lipids and controlling phospholipid homeostasis and stress response. All of these functions are essential for cell survival, especially under a stress environment. In understanding the role of SEIPIN in nLD generation, several questions remained to be resolved. For example, how does SEIPIN, as an ER protein, become localized at the inner nuclear membrane? What factors affect SEIPIN localization and translocation? Moreover, which partner proteins of SEIPIN participate in nLD generation?

SEIPIN is still a mysterious protein with many biochemistry functions waiting to be uncovered. Understanding the regulatory role of SEIPIN in LD biology and lipid storage is essential for the treatment of related human diseases (such as lipodystrophies, fatty liver, and obesity).

## Abbreviations

LD	LD
cLD	cytoplasmic LD
nLD	nuclear LD
TAG	triacylglycerols
ER	endoplasmic reticulum
ACSL	acyl-CoA synthetase long chain family member
SCD	stearoyl-CoA desaturase
GPAT	glycerol-3-phosphate acyltransferase
DGAT	diacylglycerol-O-acyltransferase
HNE	4-hydroxynonenal
FA	fatty acid
PFOA	perfluorooctanoic acid
CCTα	CDP-choline diacylglycerol phosphotransferase α
FABP	fatty acid binding protein
SERCA	sarco/endoplasmic reticulum Ca^2+^-ATPase
LIPIN	phosphatidic acid phosphatase
AGPAT	acylglycerol-phosphate acyltransferase
REEP	reticulon-like protein
